# The Response of Aboveground Net Primary Productivity of Desert Vegetation to Rainfall Pulse in the Temperate Desert Region of Northwest China

**DOI:** 10.1371/journal.pone.0073003

**Published:** 2013-09-03

**Authors:** Fang Li, Wenzhi Zhao, Hu Liu

**Affiliations:** Linze Inland River Basin Research Station, Key Laboratory of Inland River Basin Ecohydrology, Cold and Arid Regions Environmental and Engineering Research Institute, Chinese Academy of Sciences, Lanzhou, China; DOE Pacific Northwest National Laboratory, United States of America

## Abstract

Rainfall events can be characterized as “pulses”, which are discrete and variable episodes that can significantly influence the structure and function of desert ecosystems, including shifts in aboveground net primary productivity (ANPP). To determine the threshold and hierarchical response of rainfall event size on the Normalized Difference Vegetation Index (NDVI, a proxy for ANPP) and the difference across a desert area in northwestern China with two habitats – dune and desert – we selected 17 independent summer rainfall events from 2005 to 2012, and obtained a corresponding NDVI dataset extracted from MODIS images. Based on the threshold-delay model and statistical analysis, the results showed that the response of NDVI to rainfall pulses began at about a 5 mm event size. Furthermore, when the rainfall event size was more than 30 mm, NDVI rapidly increased 3- to 6-fold compared with the response to events of less than 30 mm, suggesting that 30 mm was the threshold for a large NDVI response. These results revealed the importance of the 5 mm and 30 mm rainfall events for plant survival and growth in desert regions. There was an 8- to 16-day lag time between the rainfall event and the NDVI response, and the response duration varied with rainfall event size, reaching a maximum of 32 days. Due to differences in soil physical and mineralogical properties, and to biodiversity structure and the root systems' abilities to exploit moisture, dune and desert areas differed in precipitation responses: dune habitats were characterized by a single, late summer productivity peak; in contrast, deserts showed a multi-peak pattern throughout the growing season.

## Introduction

Rainfall is a major driver of metabolism in water-limited ecosystems [1]. In desert regions, rainfall events can be characterized as rainfall pulses with discontinuous, highly variable, and largely unpredictable frequency and intensity [2] which can trigger a cascade of ecosystem responses that affect plant nutrient, water and carbon cycling [3]–[6]. One consequence of such a response is a change in aboveground net primary productivity (ANPP) [7]. In addition, climate models predict an increase in precipitation variability, which will be characterized by more extreme precipitation events punctuated by longer intervening dry periods [8], and exactly how ecosystems will respond to this change is an important question.

ANPP is a key parameter of the ecological processes that are limited by the water availability in desert regions. Soil moisture rises rapidly in these regions, and ANPP significantly increases after desert ecosystems absorb the water provided by large rainfall events [9]. Rainfall pulses can therefore significantly influence the structure and function of desert ecosystems [2], and analysis of the responses of ANPP to these pulses is critical to understanding the response mechanisms that contribute to the sustainability of desert ecosystems.

A variety of models of primary productivity response to rainfall variability-the two-layer hypothesis [10], the pulse-reserve hypothesis [2], and the threshold-delay model [11]-continue to be used with various theoretical (and subsequently operational) modifications in order to generate different scales of plant response. The two-layer hypothesis considers two important plant functional types (FT) and predicts that woody and herbaceous plants are able to co-exist in savannas because they utilize water from different soil layers (or depths), but does not consider the complex relationship of plant root system dynamics or water ascension [12]. The pulse-reserve model addresses the response of individual plants to precipitation and predicts that there are biologically important rain events that stimulate plant growth and reproduction, but this model does not account for potential delayed responses of plants to rainfall, nor explicit precipitation thresholds. Ogle [13] integrated the ideas of resource partitioning, plant delays, precipitation thresholds, and plant FT strategies into a simple threshold-delay model, but its shortcoming is that it is empirical rather than mechanistic; thus the model is difficult to scale up from individual sites, although many scientists have used it effectively to evaluate plant response [3], [13].

The response of plants to precipitation pulses has been studied extensively, from individuals to ecosystems. The research on the response of individual plants to rainfall pulses has focused mainly on the physiological and ecological parameters including sap flow, evapotranspiration, photosynthesis, respiration, and water use efficiency in arid regions. Grasslands are highly responsive to extreme precipitation patterns, and studies of these ecosystems have been carried out mostly in North American, using a variety of field experiments that involve the manipulation of rainfall timing, size and frequency [14]. Ecosystem responses to the rainfall regime have included altered ANPP [15]–[19], gas exchange [4], [20]–[22], and ecosystem respiration [23]–[25]. Some of the important conclusions about ANPP have been: Lauenroth et al. [26] discovered that the best response to individual precipitation event size of ANPP for short grass prairie was 15–30 mm. Productivity responses to more extreme rainfall regimes varied among grasslands distributed along a regional rainfall/productivity gradient, where changes in ANPP ranged from an 18% reduction to a 70% increase, depending on site [6], [15], [18]. Ecosystems in semi-arid and arid regions were much more adaptive to extreme precipitation patterns than were well-watered ecosystems like mesic tall grass prairie [15], [18], [24], [27], [28]. The result of natural ecosystem productivity responses to extreme precipitation regimes varying water conditions ultimately trends, though, to become homogenized at a large regional scale [29].

To determine the effect of precipitation pulses on an arid desert ecosystem, we selected 17 independent precipitation events during the summer from 2005 to 2012 in the middle reach of the Heihe River, and evaluated desert and dune ecosystems responses using the threshold-delay model and statistical analysis. We addressed the following questions: 1) what rainfall threshold would cause the desert ecosystem productivity to change? and 2) could rainfall pulse cause multiple productivity peaks in the desert and dune ecosystems during the growing season? Understanding responses to variation in rainfall event size and frequency will assist in assessing how desert ecosystems may change under future scenarios of more extreme precipitation regimes.

## Materials and Methods

### Ethics Statement

The weather station and study area belong to Linze Inland River Basin Research Station (39°21′ N, 100°07′ E, 1389 m), a department of the Cold and Arid Regions Environmental and Engineering Research Institute, Chinese Academy of Sciences. The study was approved by the Cold and Arid Regions Environmental and Engineering Research Institute, Chinese Academy of Sciences.

### Study Area

**Figure 1:**
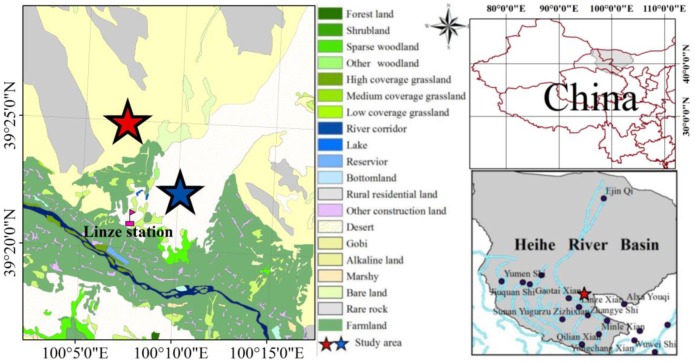
Map of the study area and location in China.

The study area (Figure 1) has two habitats – dune and stony desert ecosystems – located in a desert-oasis ecotone (39°21′ N, 100°07′ E, 1389 m) in the middle of the Heihe River Basin, in northern Linze county of Gansu province. The climate is arid to semiarid temperate continental desert. Mean annual temperature is 7.6°C with an average low of –27.3°C in January and a high of 39.1°C in July. Precipitation is highly variable in amount and spatial distribution from one year to the next [30], averaging approximately 116.8 mm/y, with 65%, on average, falling during the summer months (July-September). However, the potential evaporation is 2390 mm/y, which is 20 times the precipitation. Relative humidity is 46%. Wind direction is mainly from the northwest, and the wind speed averages 3.2 m/s, with frequent gales (wind speed ≥21 m/s). Plant distribution at both sites is patchy with variable soil properties (Table 1).

**Table 1:** Characteristics of vegetation and soil.

Characteristics	Desert	Dune
Topography	Flat	Longitudinal dune, 3–5 m
Vegetation height (cm)	19	67
Vegetation coverage (%)	12	24
Vegetation composition	*Reaumuria soongorica, Nitraria sphaerocarpa*, and a few annual plants	*Calligonum mongolicum, Haloxylon ammodendron, Nitraria sphaerocarpa* and many annual plants in August
Soil type	Aridisols	Entisols
Sandy-silt-clay (%)	77-14-9	87-9-4 [57]

### Data Collection and Processing

For this study, we used 8 years of time series growing-season NDVI datasets to evaluate patterns of plant growth at these two locations. MODIS Terra and Aqua surface reflectance 8-day composited (which reduces the influence of weather conditions and clouds) collection 5 level 3 global at 250 m spatial resolution (MOD09Q1 and MYD09Q1) in HDF format, were acquired between 2005 and 2012 in the growing season, from the following website: http://ladsweb.nascom.nasa.gov/data/search.html which is maintained by the NASA Land Processes Distributed Active Archive Center at the USGS. The images were treated with projection transformation and image registration and were clipped using a study area border executed in ENVI 4.7 Software.

MOD09Q1 and MYD09Q1 include spectral bands of red and near-infrared that are used to obtain NDVI which provides information on plant density and growing conditions on the ground [31], and it is widely used as a proxy for biomass production [32]–[34]. Because NDVI values for an 8-day period could have been recorded on any date with in that period, we assumed that the NDVI value corresponded to the middle day of the NDVI compositing period [35], when estimating response duration periods.

Meteorological data were measured by an open-field weather station located approximately 5 km from the study sites; the meteorological variables were wind speed, air temperature, relative humidity, net and photosynthetically active radiation, soil temperature, rainfall, and atmospheric pressure. These parameters were measured using an AG1000 automatic weather station (Onset Computer Corporation, Pocasset, MA, USA). The sensors were installed at two levels above the ground (2 and 3 m). Rainfall was measured with a tipping-bucket rain gauge (model TE525, metric; Texas Electronics, Dallas, TX). The meteorological data were measured at a frequency of 10 Hz and recorded every 5 min using a CR1000 datalogger (Campbell Scientific Inc., Logan, UT), then stored as 30-min mean data, whereas rainfall and wind data were stored as 10-min mean data.

Continuous rainy-day data observed at the Linze meteorological station were treated as independent rainfall events. Those with less than 5 mm were removed, as this was considered to be ineffective rainfall. We then selected 17 independent precipitation events according to the following three standards [36]: (1) no other major rainfall events occurred during the preceding 15 days, the antecedent soil moisture was relatively stable, and by 15 days after the pulse, the soil moisture returned to pre-pulse conditions [37]; (2) the event occurred toward the middle of the growing season (June-August), when temperature was relatively high; and (3) the event was distributed evenly at a particular rainfall class. Seventeen precipitation events were selected (Table 2).

**Table 2:** Choice of precipitation events.

Eve-nt	Start time	Continuous days (d)	The rainfall event size(mm)	NRDBRE(d)	NRDARE(d)	Response duration (d)
1	06-7-06	3	20.8(11;9.2;0.6)	>30	12	5–8
2	06-7-21	1	17.7	12	10	6–8
3	06-7-31	2	6.7(1;5.7)	9	11	8
4	06-8-18	5	9.8(0.3;9.5)	11	>30	desert (9),dune(17)
5	07-6-15	3	14.8(5.6;8;1.2)	57	29	7
6	07-7-04	2	7.4(6.2;1.2)	16	11	9
7	07-7-17	5	43.4(3.5;6.2;27.2;1.8;4.7)	11	42	23-26
8	07-9-02	1	12.3	13	>20	5-8
9	08-7-29	2	24.2(11.8;12.4)	15	52	desert (7),dune(20)
10	09-8-18	1	23.5	>20	17	desert(10),dune(17)
11	10-5-25	1	16	10	>20	8
12	10-7-01	2	9.8 (0.1;9.2)	36	27	5-9
13	11-6-25	5	30(5.0;2.6;0.8;10;0.2;11.4)	>60	19	8-10
14	11-7-23	1	6.4	19	18	4–8
15	11-8-10	5	37.2(1.4;5;13.6;3.8;12.4)	18	40	13–19
16	12-6-05	2	18.4(15.8;2.6)	>20	19	desert (9),dune(17)
17	12-7-29	1	12	12	14	12

NRDBRE: No-rain days before rainfall events; NRDARE: No-rain days after rainfall events. Like “20.8 (11; 9; 2; 0.6)” in the rainfall event size column means the continuous four rainy days' data observed and we treated as independent rainfall size.

### Statistical Analysis

**Figure 2:**
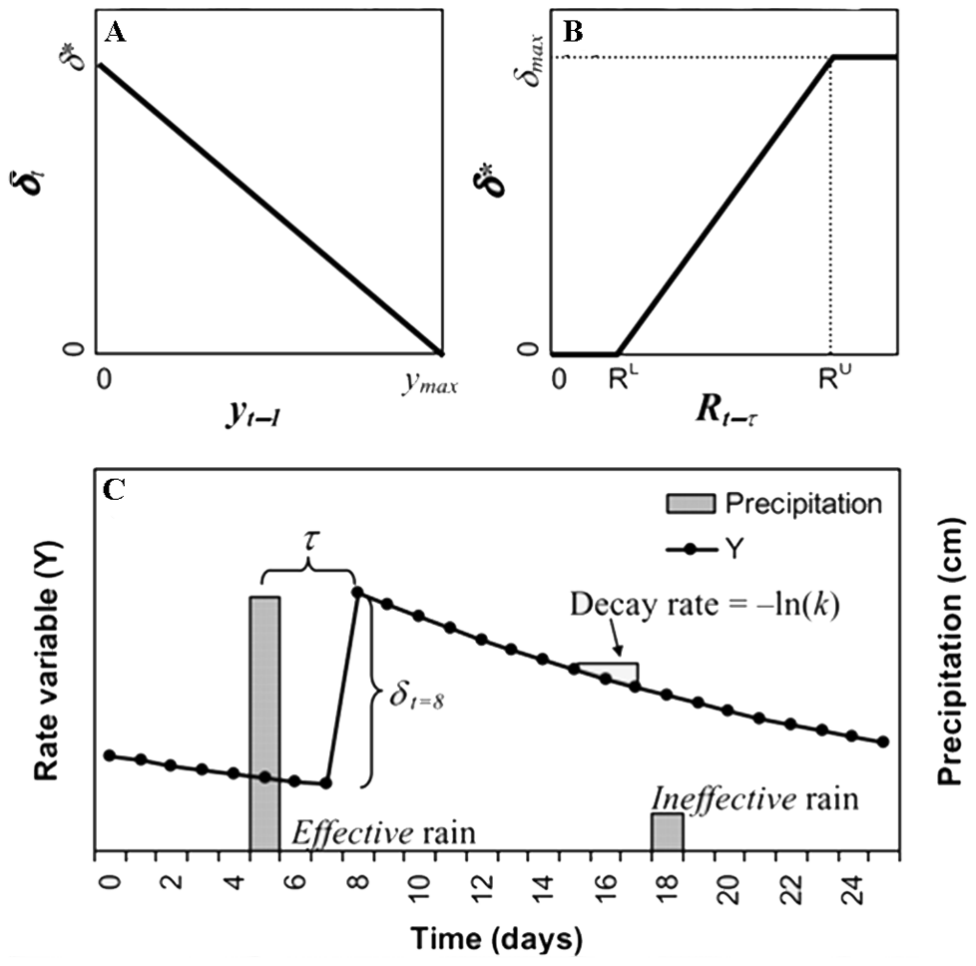
A Conceptual diagram of the threshold-delay model based on Eqs. 1, 2, and 3 [11]. (A) is the relationship between δt (the magnitude of the increase in the response) and yt–1 (the previous state of the response variable), where ymax is the maximum potential value of the response variable and δ* is the maximum potential response increase; (B) is the relationship between δ* and rainfall size at lag τ (days). RL is the lower threshold below which rain events do not stimulate a response. RU is the upper threshold above which rain events than do not yield additional benefits; and (C) provides a hypothetical response curve.



We used the threshold-delay model [11] (Figure 2) to analyze and compare the response of desert and dune ecosystems to precipitation pulses, including speed of the response, magnitude of the response, duration of the response and the thresholds below or above which no response, or no further response was observed. The threshold-delay model is an integrative framework for plant growth delays, precipitation thresholds, and plant FT strategies, which captures the nonlinear nature of plant responses to rainfall pulses. It rests on two assumptions: that there exist lower and upper thresholds on the size of a precipitation pulse to stimulate plant FTs response and that the magnitude of the response has upper limits. It is based on six parameters including lower and higher precipitation thresholds (*R^L^ or R^U^*), lags (*τ*), potential responses (*δ_max_*), maximum response rates (*y_max_*), and the reduction in the response variable over time (*k*). The model can be expressed as follows:

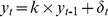
(1)

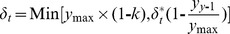
(2)

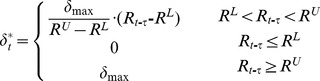
(3)where *y_t_* is the response variable, *y_t−1_* is the antecedent value of this variable, *y_max_* is the maximum response variable value, *δ_t_* is the magnitude of the response increase, δt* is the potential response increase, *δ_max_* is the maximum potential response increase, *R^L^* is the lower threshold of rainfall, *R^U^* is the upper threshold of rainfall, *R_t−τ_* is the effective rainfall event, τ is the time lag, t is the response time, and k is the reduction rate.

We identified the rainfall threshold using a linear regression model of rainfall size and NDVI increments induced by different rainfall event size, using Origin software, version 8. When NDVI increment was equal to zero, the corresponding rainfall size was determined as the lower precipitation threshold. When NDVI increments increase only negligibly or even decreased slightly, the corresponding rainfall size was used as the upper threshold (due to the limit of large rainfall size events, it is difficult to determine the maximum threshold of precipitation). The other parameters of threshold-delay were determined by multiple linear regression based on Eqs.1, 2, and 3 [3], [13]. Model parameters are the average value of 15 rainfall events pulses response (two 5–10 mm rainfall events were removed from the analysis), except for *R^L^, R^U^, δ_max_. τ* was estimated. The parameters determined the overall response process of desert and dune habitats, to different sizes of rainfall events. Response durations were estimated from the time intervals between the dates of first documented NDVIs response after rainfall pulses and the following dates of documented maximum NDVIs.

We analyzed the significance in response of NDVI to rainfall pulses using a three-factor ANOVA to compare both the main effects and the interactive effects of rainfall event size categories, habitats (desert vs. dune), and before and after responses, using SPSS software, version 18. The rainfall event size categories were divided into five types, 5–10 mm, 10–15 mm, 15–20 mm, 20–30 mm, and greater than 30 mm, according the number and distribution of 17 precipitation event sizes. Three replicate events fell within each size category. NDVIs of after responses were selected as the maximum response variable values because the response lasted for a significant period of time. Both the Terra and Aqua satellites were used to quantify plant responses to all 17 rainfall events, and the two different estimates were treated as independent statistical replicates for a given event and ecosystem type. In addition, the error terms in this paper represent the standard errors of the all the pixels in the study area. Pearson correlation analysis was used to study the relationships between the corresponding variables. Also, the growth rate was calculated as the difference between before and after responses, divided by before responses. Similarly, the NDVIs of the after response were selected as the maximum response variable value.

## Results and Analysis

### Rainfall Pulse Characteristics

**Figure 3:**
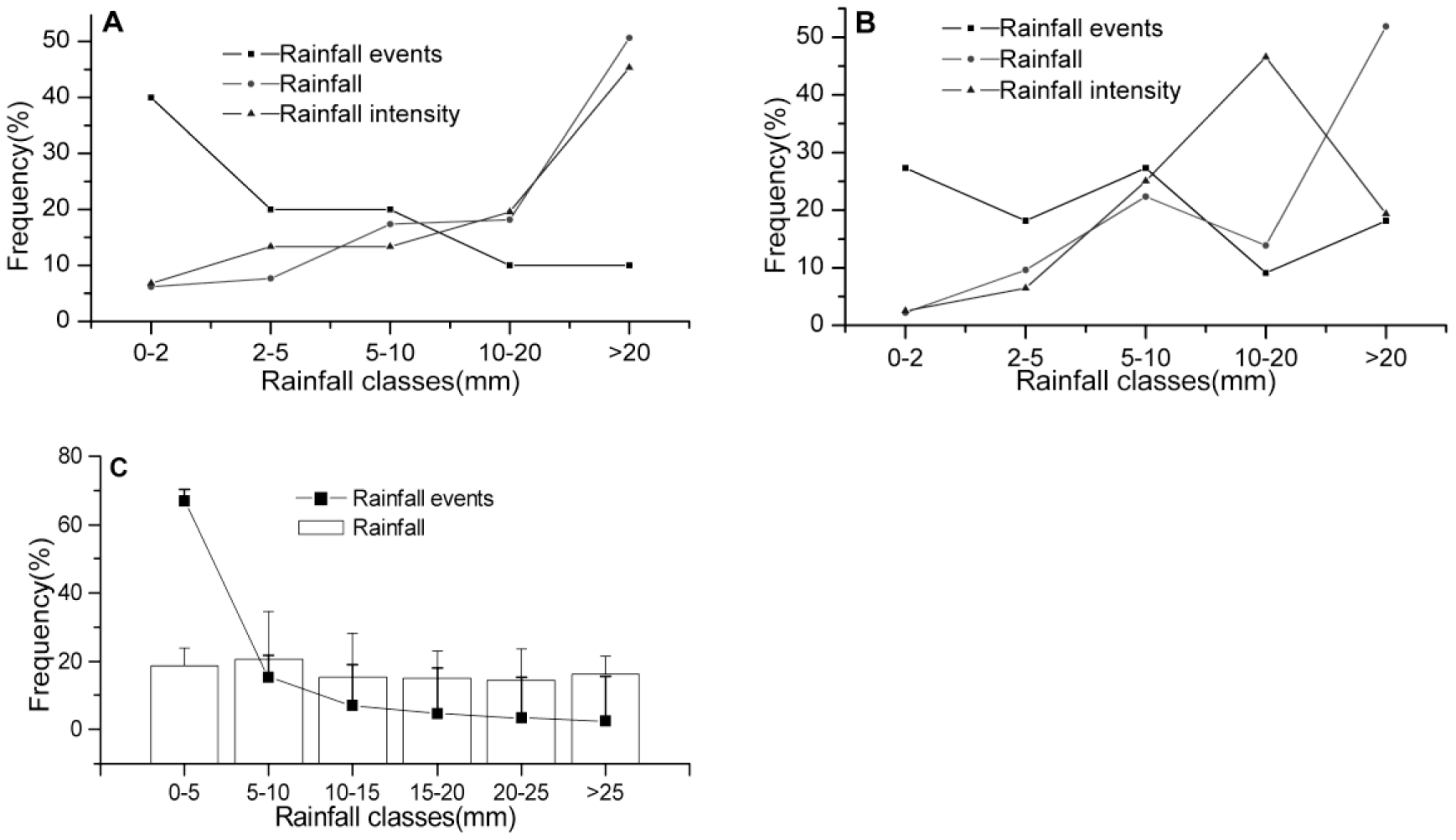
Frequency distributions of rainfall and rainfall events. (A) From April to October in 2007; (B) From April to October in 2011; (C) From April to October between 2004 and 2012.

Average annual rainfall for the study area was 124 mm from 2005 to 2012, with approximately 75% of this occurring between June and September and 25% occurring from October to May (Figure 3). Most of the rainfall events were small, with the majority (67%) being less than 5 mm. When rainfall event size increased from 5 mm to 50 mm, the frequency decreased, during the growing season. The frequency of all the different rainfall classes was similar, between 14% and 20%; the differences were reduced among the rainfall classes. All of these characteristics exhibited the rainfall pulse patterns of an arid region.

The accumulative rainfall from June to August in 2007 and 2011 were identical 83.5 mm. The precipitation patterns, however, differed between 2007 and 2011 (Figure 3b, 3c), with the majority of rainfall events in 2007 being small, but with an even distribution of rainfall events in 2011. The percentages of the total amount were similar, with the majority from large precipitation events.

### Response Process of NDVI to Rainfall Patterns in Two Habitats

Plant responses differed significantly among rainfall event size categories, habitats, and before and after responses (p<0.01; Table 3). The rainfall event size categories × habitats interaction was significant. This pattern showed that NDVI in desert ecosystems responds significantly to rainfall pulses.

**Table 3:** Statistical analysis of the changes in NDVI in response to rainfall pulses.

Factor	Df	F statistic
Rainfall classes	4	12.856***
Habitats (dune vs. desert)	1	240.083***
Before and after responses	1	8.757**
Rainfall classes × Habitats	4	3.377*
Rainfall classes × Before and after responses	4	1.902
Habitats × Before and after responses	1	0.929
Rainfall classes × Habitats × Before and after responses	4	0.298

** P<0.01,*P<0.05.

**Figure 4:**
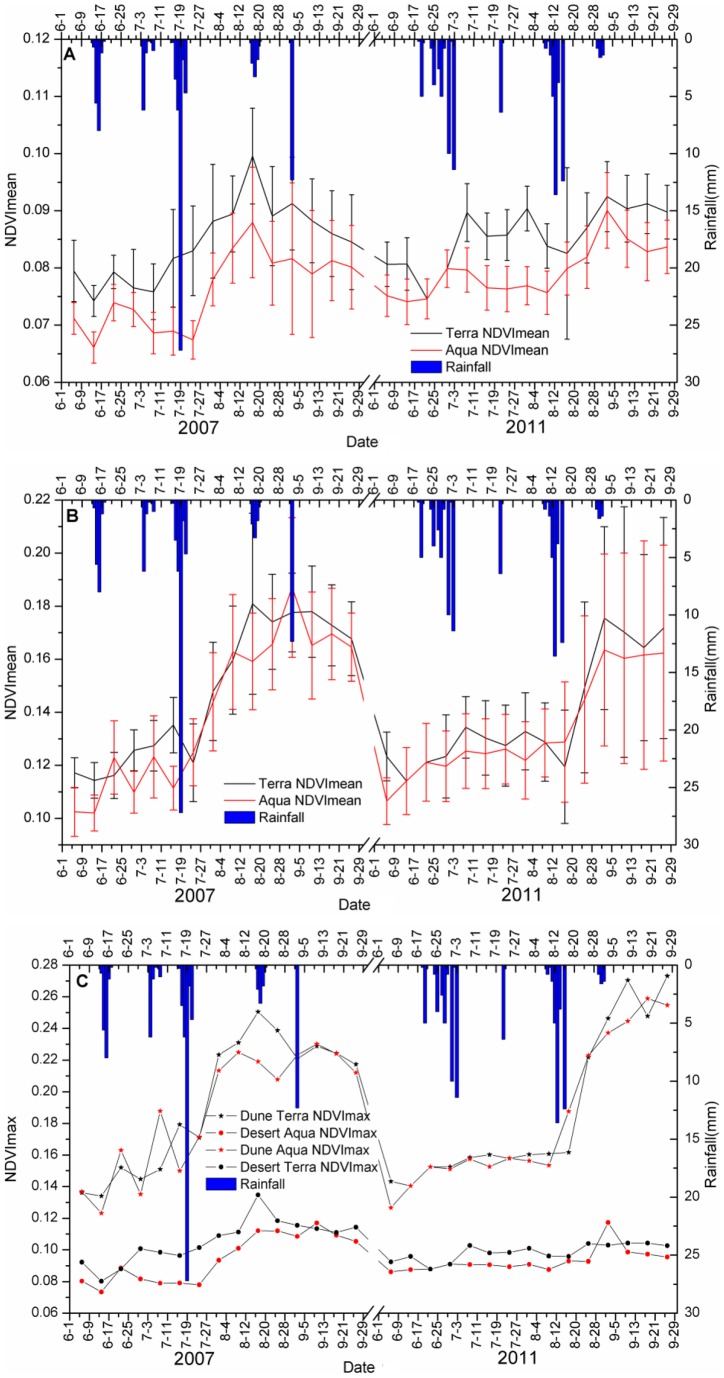
Intra-seasonal precipitation patterns in the threshold-delay data for NDVI in response to rainfall pulses. Error bars represent standard error for all the pixels in the study area. (A) The mean NDVI of the desert study area response to rainfall pulses for Terra and Aqua satellites. (B) The mean NDVI of the dune study area response to rainfall pulses for Terra and Aqua satellites. (C) The Maximum NDVI of this two-habitat study area's response to rainfall pulses for Terra and Aqua satellites.

The results indicated that the response pattern of NDVI agreed with the description proposed by the threshold-delay model (Figure 4), but the maximum rainfall threshold was not reached. The NDVI of the dune ecosystem increased rapidly with 43.7 mm of rainfall on 20 July 2007, reaching its maximum (0.18), with a 26.21% growth rate; in the desert ecosystem, the same amount of rainfall, on August 17, resulted in a maximum NDVI of 0.10, with a growth rate of 31.53%. In response to lower amounts: the NDVI in the desert and dune ecosystems responded significantly at 6.4 mm of effective rainfall on 22 July 2011, but the increases here were much smaller – 0.4% and 1.46% respectively. In both habitats, when the rainfall was less than 43.7 mm, the NDVI was monotonically increasing and the maximum rainfall threshold was not reached.

It is clear that desert ecosystems are sensitive to the rainfall regime, with responses of ANPP contingent on mean rainfall levels, although in different ways for different desert types. There was more sensitivity to growing season rainfall regimes in the dune NDVI than in the desert. In the dune region, a large rainfall event led to extended periods of soil moisture content, increased plant photosynthesis and increased NDVI of by about 30%, and peak production occurred in August, when a large number of annual plants appeared. The opposite response occurred in the desert, where a fluctuation shift to greater than 5 mm, but less than 30 mm, event size resulted in a 2%–10% increase of NDVI, and multiple peaks appeared between June and September.

Ecosystems clearly have a “memory” of past precipitation events which can last at least several decades [38]. The NDVI curves showed that vegetation response to rainfall events was not immediate, but delays or time lags occurred following the rainfall events and they ranged from 8 to 16 days. The duration of response for different precipitation events sizes varied. It was less than 20 days when rainfall fell below 25 mm, but it endured for a maximum of 32 days after 43.7 mm of rainfall. Due to the limitation of MODIS image temporal resolution in this study, the lag time and the response duration were estimated.

### Response Relationship of NDVI to Rainfall Pulses

The response of NDVI to rainfall pulse was influenced consistently by rainfall event size, duration of rainfall and maximum precipitation intensity (Table 4). Although we did not detect a significant correlation between the duration of the dry interval and the two habitats' NDVIs, the variability of duration of the dry interval could weaken the response of soil moisture to a rainfall event [39], and soil moisture is a key parameter in the rainfall-productivity relationship.

**Table 4:** The Pearson's correlation coefficients among the response increase of NDVI, rainfall event size, maximum and mean precipitation intensity, duration of rainfall and dry interval.

Habitat	Rainfall event size	Maximum precipitation intensity	Mean Maximum precipitation intensity	Duration of rainfall	Dry interval
Desert	0.93028**	0.41453*	–0.17627	0.70174**	–043
Dune	0.77873**	0.43727*	–0.16499	0.69071**	–083

** P<0.01,*P<0.05.

**Figure 5:**
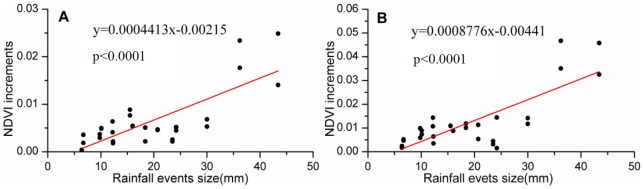
Relationship between NDVI increments and rainfall event sizes in different habitats. (A) Dune ecosystem: n = 34, adjusted r2 = 0.59, p<0.0001; (B) Desert ecosystem: n = 34, adjusted r2 = 0.60, p<0.0001. The relationships show significant linear trends for dune and desert ecosystems. The observations used for these analyses come from both the Terra and Aqua satellites. Due to the lag, here, the NDVI increment is the the maximum response to a rainfall event. The error bars represent standard errors for all the pixels in the study area.

Increments in the NDVI could be a direct response of the ANPP to different rainfall event sizes (Figure 5). The relationship between NDVI increments and rainfall event sizes was similar between the two habitats, although the dune ecosystem maintained higher overall rates of NDVI change than the desert ecosystem. The curves revealed that the increments in NDVI could be expressed as a linear function of rainfall event size. When the increment in NDVI was equal to zero, meaning that the NDVI response started after the rainfall event, the minimum rainfall threshold of the two habitats was about 5 mm. Although the fit of linear regression reached a remarkable level, the distribution of scatters reflects a threshold. When the rainfall event size was less than 30 mm, the increments of NDVI were about 0.01 and 0.005 of increase, for dune and desert respectively, but the increments reached 0.04 and 0.02 with greater than 30 mm of rainfall. However, the maximum rainfall event size for the desert ecosystems was not determined and needs further research.

**Figure 6:**
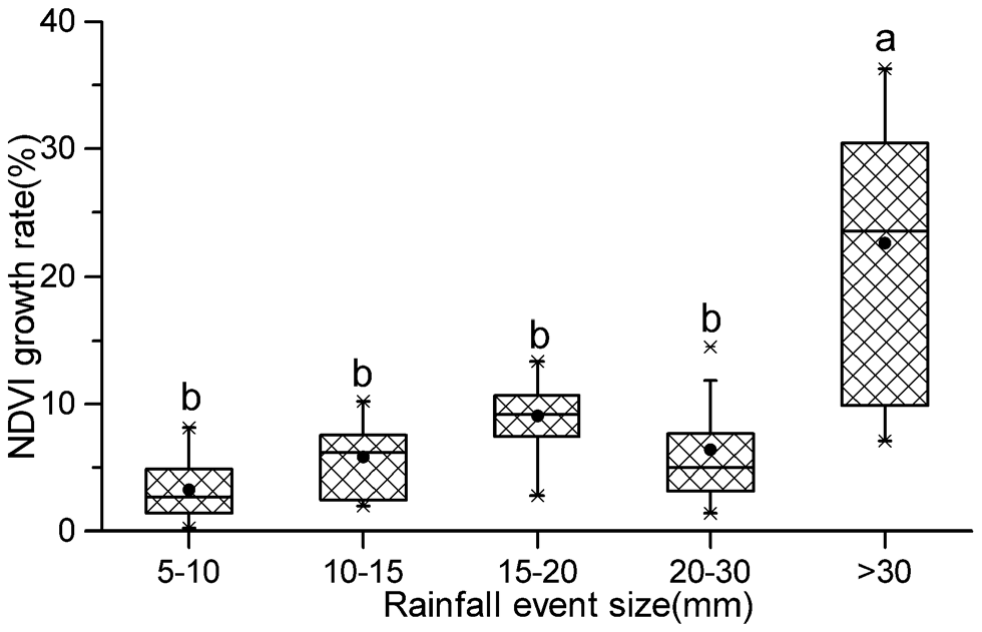
Percentage increase in NDVI, from the day before rain to the day of peak NDVI, in response to different rainfall size classes. Letters above columns represent significantly different treatments (Tukey's HSD test, P<0.05). 5–10 mm: n = 20; others: n = 12. The observations used for these analyses come from both the Terra and Aqua satellites.

The percentage increase in NDVI was significantly smaller (P<0.05) for the 0–30 mm classes than for rainfall in the >30 mm rainfall class (Figure 6). The NDVI growth rate also reflected a threshold, similar to the conclusion from Figure 4. The entire desert ecosystem NDVI growth rate was 3%–9% when rainfall was less than 30 mm, but it reached 22.5% with more than 30 mm – a 3- to 6-fold increase. Our results showed that in this study region a 30 mm rain event size is required for NDVI to increase most sharply.

We determined the rainfall threshold by means of linear regression model, and the parameters of the threshold-delay model by means of multiple linear regression (Table 5). The lower thresholds for desert and dune habitats were identical, with values of 5 mm. No upper threshold appeared; hence we could not determine the maximum potential response value (*δ_max_*). The values of *k* were different for desert and dune habitats, 0.9564 and 0.9547, respectively, indicating that the duration of desert habitat response to a precipitation pulse is greater than that for dune habitat. The magnitude of the increase in the response (*δ*_t_) and the potential response (δ_t_^*^) values of desert (dune) were 0.0065 (0.0161) and 0.0139 (0.0250), respectively, indicating that the dune response values were significantly greater than desert after the same precipitation pulses. The maximum potential response variable values (*y_max_*) were 0.1491 and 0.3554 for desert and dune habitats, respectively, that is to say, the NDVI of desert and dune can reach up to 0.1491 and 0.3554. Due to the limitation of MODIS image temporal resolution in this region, the lag times were estimated; they ranged from 8 to 16 days.

**Table 5:** The parameters of the threshold-delay model for changes in NDVI in response to rainfall pulses.

Habitat	*R^L^*(mm)	*k*	*δ_t_*	*y_t-1_*	*y_max_*	*τ* (day)	δ_t_^*^
Desert	5	0.9564	0.0065	0.0792	0.1491	10–20	0.0139
Dune	5	0.9547	0.0161	0.1267	0.3554	10–20	0.0250

*R^L^* is the lower threshold of rainfall, *k* is the reduction rate. *y_t−1_* is the antecedent value of the NDVI, *δ_t_* is the response increase, *τ* is the time lag, *y_max_* is the maximum response variable value, and δ_t_* is the potential response increase.

## Discussion

As was hypothesized, the use of an NDVI dataset extracted from a MODIS image dataset with 8-day temporal resolution and 250 m spatial resolution revealed the rainfall-productivity relationship at the ecosystem scale, but the relationships differed in the temporal scale. In the scale of years, NDVI variation during the growing season was closely related to the summer cumulative precipitation and also to the previous year's fall and winter precipitations, indicating that in desert ecosystems winter rainfall may sometimes be more important to plant FT response than is summer rainfall [11], [40]–[42]. However, temporal variation in ANPP at the local scale is controlled by a variety of interacting factors, mainly the seasonal and spatial variability of precipitation event pulses [43]. In fact, research has shown that high rates of shrub production are triggered by water pulses during warm periods [44].

### Response of the Desert Ecosystem ANPP to Rainfall Pulses

Precipitation is unimodal, with the majority of the precipitation occurring in the summer and most of the rainfall events being small, 67% of them less than 5 mm (Figure 3), making the rainfall events that produced the eco-physiological responses particularly important. Our results showed that a precipitation event size of more than 5 mm does have a pronounced effect on the NDVI of the desert ecosystem independent of precipitation amount (p<0.001) (Table 3). Different rainfall classes present significant difference, a result that support the hierarchical response, but a 30 mm rainfall event is the threshold with a large response (Figure 6).The effect of rainfall size was much larger than the dry interval of rainfall events (Table 4), a result consistent with the conclusion that the precipitation event size is most important at the driest site considered, and the spacing of events most important at the mesic site [15], [45], [46].

**Figure 7:**
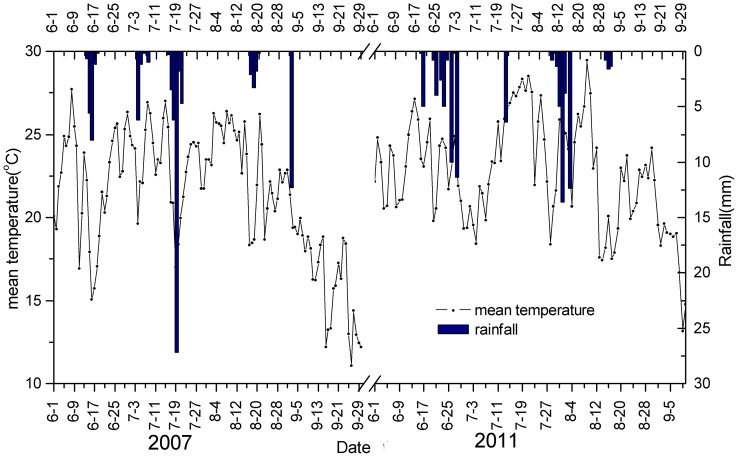
Patterns of mean temperature in response to rainfall pulses.

Linear regression model suggested that at least one precipitation event, close to or above 5 mm, was required for both desert and dune ecosystems' NDVI increased threshold value to be reached during the growing season (Figure 5). On average, therefore, rainfalls supplying more than 5 mm were likely to be associated with productivity in this ecosystem, despite a slight decrease in average temperature following rainfall (Figure 7). This rainfall threshold represents an ecologically significant rainfall event that interacts with a plant's water-use patterns of utilizing soil moisture pulses at particular infiltration depths or durations [9], [47]. For example, in the North American short-grass steppe, it has been shown that events as small as 5 mm improved water conditions and increased soil water potential [48]–[50]. Furthermore, for the shrubs *Nitraria sphaerocarpa* and *Elaeagnus angustifolia* in a desert ecosystem, the lower stem rainfall thresholds were 5 mm for the sap flow response [13], an indicator of the potential for shrub growth and water use patterns, whereas in a temperate Australian woodland, the threshold needed for rain events to elicit an increase of sap flow exceeded 20 mm [3]. Meanwhile, in grass (shrub) communities, spring and summer precipitation thresholds for CO_2_ uptake were 23(59) mm and 51–148(57–140) mm, respectively, and the spring response had an impact on the summer threshold values [42]. Hence it can be concluded that a rainfall event of 5 mm is an ecologically significant rainfall event for ANPP responses in desert ecosystems.

Many biological state changes where organisms transition from a lower to a higher state of physiological activity, require a minimal triggering event size [9]. In this study, the threshold needed for a sharply higher NDVI response was 30 mm, past which rates of NDVI growth increased more than 3- to 6- fold (average of 6% and 22.5% NDVI for rainfall <30 and >30 mm, respectively) (Figures 5 and 6). Compared with a 5 mm rain event, shrub species with deep roots would continue to take up water from the deeper soil after a large event [9], [12], which could trigger a large productivity increase. For example, at the season scale, Sponseller et al. [44] indicated that rates of stem growth with a threshold of over 100 mm summer precipitation increased more than eightfold. Compared to previous research in grassland, this value is much higher than the one for a short-grass steppe, in which precipitation events of 15 mm to 30 mm contributed most of the effect of precipitation on ANPP [26]. This difference was most likely the result of higher demand for water in the desert ecosystem, caused by lower holding capacities and hydraulic conductivity, higher evaporation rates, and different soil moisture dynamics [43]. In addition, biodiversity structure and the ability of root systems to exploit moisture at varying depths differed greatly between these two ecosystems [20].

We used the threshold-delay model to analyze the desert and dune ecosystems' response to each precipitation pulse, for 15 events, and ultimately estimated several model parameters which quantitatively demonstrate the response process. The parameter *k* represented complex interactions among root profiles, density, structure and morphology and soil water dynamics [11]. In this study, the desert's and dune's *k* values were similar but the desert's was slightly greater (Table 5), because *Reaumuria soongorica* is a dominant and constructive species whose root system is relative deeper, with lower density. Also, the soil water potential was higher in desert habitat. In contrary, the root system of *Nitraria sphaerocarpa*, a dominant species in dune habitat, is mainly horizontal, and there is an added impact of annual plants in late growing season, resulting in a smaller *k* value for the dune, a result consistent with the trend found by Ogle et al. [11] and Zeppel et al. [3] that the *k* values of annual plants and shallowly rooted woody plants are smaller. The parameter *y_max_* reflects differences in physiology and growth strategies [11]. The maximum potential response variable values (*y_max_*) in this study were 0.1491 and 0.3554 for desert and dune habitats, respectively, indicating that the physiology and ANPP response levels of dune plants to precipitation pulses were much higher, because of biodiversity and structure differences, in particular for annual plants whose responses occurred in late growing season, and whose root systems were better able to exploit moisture.

Time lags may vary among species and regions [50], [51], and may be influenced by other factors, such as slow infiltration of rainfall to soil layers where active roots reside. These lags may also be proportional to the intensity of drought [52]. The lag time for response averaged about 8 to 16 days, the lack of precision being due to the limitation of MODIS image temporal resolution in this region, as well as with the multi-species environment. The response duration varied with the rainfall event size. Within morphologically similar species, it was determined by the variation in tolerance to soil water potential [9]. In this study, the differences in response duration were not obvious between dune and desert ecosystems.

### Different Responses of the Two Habitats' ANPP to Rainfall Events

The response pattern showed that NDVI varied predictably between dune and desert habitats, based on rainfall event size (Figure 4). Dune habitats were characterized by a single, late summer productivity peak after two large rainfall events of about 43.4 mm and 37.2 mm; in contrast, deserts showed a multi-peak pattern during the growing season. Furthermore, the CO_2_ uptake of shrubs in response to precipitation events with multiple peaks in a growing season when rainfall distribution is suitable [42], is a trait consistent with the multi-peak model in ANPP.

These differences in NDVI responses between the two habitats were likely the result of the species composition and lower diversity of the desert, which is mainly semi-shrub, compared to those of the dune, as well as of the appearance of many more annual plants in August (Table 1). The structure of the vegetation, ranging from plant density to species composition, determines the density of meristems where plant growth occurs, and so may provide corresponding fluctuations in production in response to fluctuations in precipitation [52]. Multi-peak time biomass in desert habitat was more obvious to be observed in a precipitation regime with temporal distribution uniformity of relatively even and large precipitation events in the growing season. These results indicate that the temporal distribution of rainfall events strongly regulates periods of biological activity in desert and dune ecosystems, a result which agrees with the study of Heisler-White et al. [16]. Moreover, winter or early spring precipitation establishes deeper soil moisture content at the onset of the growing season and frequently represents a peak in the entire growing season ANPP dynamics, favoring shrubs [42].

NDVI responses were different in the early and middle growing seasons (Figure 5) in 2011. At the event on 25 June 2011, the dune ecosystem had no apparent increase of NDVI, but the desert did. This difference is likely related to life forms and functional groups, and responses to abiotic drivers [43] to which sub-shrub and small shrub species in the desert are sensitive, whereas shrub species in the dune ecosystem evidently are not. This result also indicates that the time of precipitation is important to NDVI, because of the physiological state of shrubs. At the start of the summer precipitation, the threshold is higher, and productivity is lower for the shrub [42] and the variation in soil moisture storage and carbon and water fluxes among species, soil type and precipitation regime treatments were minor compared to the variation observed for these factors in ecosystem fluxes following the August precipitation pulses [53]. In addition, adequate precipitation during the previous winter and spring may supply moisture to the deeper roots of shrubs, allowing them to aggressively exploit water availability with their physiologically active state during the early growing season [54]. In 2010, fall and winter precipitation was 93.6 mm, which greatly activated NDVI in the early growing season; hence although the summer rainfall was smaller in 2011 than in 2007, yet the NDVIs during the growing seasons were almost identical.

## Conclusion

Arid regions are prone to drought because annual rainfall accumulation depends on a few effective rainfall events [55]. This research focused on understanding how a desert ecosystem responds to changes in precipitation regimes. Our results showed that the response of NDVI to rainfall pulse begins at about 5 mm, and that when precipitation is above 30 mm, NDVI rapidly increases 3- to 6-fold, demonstrating the importance of 5 mm and 30 mm rainfall events for plant survival and growth in desert regions. In addition, in the dune ecosystem there is a precipitation response with a single biomass peak, in August, but in the desert the peak times for biomass are in June-September, especially when the distribution of precipitation events is even. These differences result from the corresponding differences in soil properties and vegetation composition.

Assessing the impact of precipitation variability on ecosystem productivity and function is inherently difficult due to the spatial and temporal differences within a site as well as across an entire region [56]. This study had some limitations: 1) sufficient effective precipitation events are rare in arid regions and differences in the July-August response varied with environmental variables; 2) physiological states affect results: the summer precipitation threshold starts later, the threshold itself is higher, and shrub productivity is lower in the dune ecosystem [42]; 3) the study likely does not reflect the more dynamic responses of desert ecosystems. Despite these limitations, though, the conclusions from this research can provide a reference for field control rainfall experiments and can also contribute to the sustainability of desert ecosystems.
